# Genomic Promoter Analysis Predicts Functional Transcription Factor Binding

**DOI:** 10.1155/2008/369830

**Published:** 2008-10-30

**Authors:** J. Sunil Rao, Suresh Karanam, Colleen D. McCabe, Carlos S. Moreno

**Affiliations:** ^1^Department of Epidemiology and Biostatistics, Case Western Reserve University, Cleveland, OH 44106, USA; ^2^Department of Pathology & Laboratory Medicine and Winship Cancer Institute, Emory University School of Medicine, Atlanta, GA 30322, USA

## Abstract

*Background*. The computational identification of functional transcription factor binding sites (TFBSs) remains a major challenge of computational biology. *Results*. We have analyzed the conserved promoter sequences for the complete set of human RefSeq genes using our conserved transcription factor binding site (CONFAC) software. CONFAC identified 16296 human-mouse ortholog gene pairs, and of those pairs, 9107 genes contained conserved TFBS in the 3 kb proximal promoter and first intron. To attempt to predict in vivo occupancy of transcription factor binding sites, we developed a novel marginal effect isolator algorithm that builds upon Bayesian methods for multigroup TFBS filtering and predicted the in vivo occupancy of two transcription factors with an overall accuracy of 84%. *Conclusion*. Our analyses show that integration of chromatin immunoprecipitation data with conserved TFBS analysis can be used to generate accurate predictions of functional TFBS. They also show that TFBS cooccurrence can be used to predict transcription factor binding to promoters in vivo.

## 1. Background

One of the important challenges in computational biology is the accurate prediction of functional transcription factor binding sites (TFBSs). A primary reason that accurate prediction of relevant TFBS remains difficult is due to the short (6–12 bp) degenerate motifs represented as position weight matrices (PWMs) that match high numbers of false positives in genomic sequences. We previously described the conserved transcription factor binding site (CONFAC) software that uses a comparative genomic approach to identify evolutionarily conserved and statistically overrepresented TFBS [1]. The use of comparative genomics to identify functional TFBS [2–9] is based on the hypothesis that functional noncoding genomic sequences are more highly conserved during evolution than nonfunctional TFBS.

Here we have applied the CONFAC analysis to the complete set of 21222 RefSeq transcripts identified in the Human Genome. We mined our conserved TFBS data in combination with public in vivo occupancy data [10] using Bayesian methods to determine the sequence contexts that influence binding of the HNF1 and HNF4 transcription factors. We predicted the binding of HNF1 and HNF4 to promoters in human pancreatic islet cells and hepatocytes in an independent test set of 1349 genes with 84% accuracy.

## 2. Results

We have applied CONFAC to the complete set of 21222 publicly available human RefSeq transcripts. CONFAC works by identifying the conserved sequences in the 3 kb proximal promoter region and first intron of human-mouse ortholog gene pairs and then identifying TFBS, defined by position weight matrices from the MATCH software [11], that are conserved between the two species [1]. Conserved sequence is defined as that which is aligned between the human and mouse genomes by pairwise BLAST of the 3 kb upstream and first intron. Thus, those sequences are not necessarily aligned within the global human-mouse genome alignment. CONFAC then identifies within those conserved sequences the TFBS that fall within a 25 bp window of each other in the human- and mouse-conserved sequences. The cutoff threshold parameters for core similarity score (CSS) and matrix similarity score (MSS) used were 0.85 and 0.75, respectively. The TFBSs that meet these criteria are defined here as conserved TFBSs. Of the 21222 available human RefSeq transcripts, CONFAC identified 16296 human-mouse ortholog gene pairs, and of those pairs, 9107 genes contained conserved TFBS in their promoter regions. We observed conserved TFBS for 310 position weight matrices (PWMs) from this initial analysis. The result was a 9107-gene by 310-TFBS table in which each element represented the number of conserved occurrences of each TFBS in the promoter of each gene. The complete dataset from this analysis is provided in the supplementary material (see additional file 2: SuppTableS1.txt for the complete dataset available online at doi:10.1155/2008/369830), and is also available on our website (http://morenolab.whitehead.emory.edu/pubs/Refseq/).

### 2.1. In Vivo Occupancy Predictions

**Figure 1:**
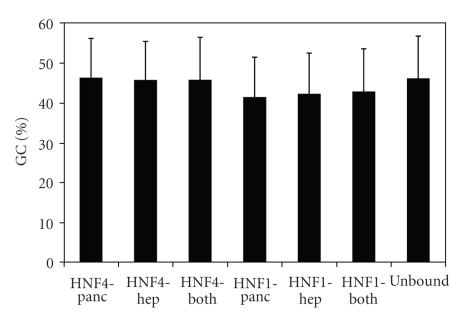
%GC content of the conserved promoter sequences in each of the seven groups considered. Plotted are the mean and standard deviation of the %GC in each promoter set. Although there is slightly higher GC content in the HNF4-bound groups, no statistically significant GC bias was observed for any of the groups analyzed for patterns of conserved TFBS.

We sought to develop methods to enable integration of in vivo occupancy data from ChIP-chip studies with our genomic CONFAC TFBS analysis to determine what TFBS patterns might improve computational prediction that a TFBS would be bound in vivo. Towards this end, we utilized publicly available ChIP-chip data on HNF4 and HNF1 [10] to analyze the patterns of TFBS identified by CONFAC. We classified the genes from this dataset into seven groups: those (1) bound by HNF1 in hepatocytes, (2) bound by HNF1 in pancreatic islets, (3) bound by HNF1 in both tissues, (4) bound by HNF4 in hepatocytes, (5) bound by HNF4 in pancreatic islets, (6) bound by HNF4 in both tissues, and (7) unbound genes. The public in vivo occupancy data for HNF1 and HNF4 [10] on 13046 genes was cross-referenced with the 9107 genes containing conserved TFBS. A total of 6683 genes were present on the ChIP-chip microarray and had conserved TFBS data using CONFAC. This set of 6683 genes was used for further analysis. CONFAC uses a pairwise-BLAST method for generation of conserved sequence alignments between promoters of human-mouse ortholog pairs. This approach allows for local alignments as opposed to global genome alignments. However, one limitation is that if no significant alignment is found, there is no sequence to analyze for TFBS, no matter what parameters for core and matrix similarity are utilized. Complete data on conserved TFBS for each of the seven groups analyzed are available in the supplementary material (see Additional Files 3–9: Supplementary Tables S2–S8). To ensure that our TFBS data was not skewed by differences in GC-content, we examined the %GC content of the conserved promoter sequences in each of the seven groups of genes, and found that each group had a similar %GC content in its conserved promoter sequences (Figure 1).

Previous studies have utilized Bayesian methods to identify TFBS combinations that might be predictive of gene expression patterns [12]. For our analysis, we employed the Bayesian Analysis for Microarrays (BAMarray) software [13]. We used BAM to generate pattern-specific lists of significant TFBS where each pattern type corresponded to each of the seven groups of genes described above. This dataset has been mined in other studies using multivariate adaptive regression splines to identify cooccurring TFBS pairs that correlated with expression patterns and localization data [14]. However, these earlier analyses identified patterns correlated with occupancy, and did not attempt to predict whether a promoter was occupied or unoccupied in a blinded fashion. Moreover, BAM estimates are model-averaged, and have been shown to have lower mean squared error than competing nonmodel-averaged estimates [15], thus resulting in more reproducible TFBS pattern sets.

We trained the BAMarray software using 80% of the genes that fell into each of these seven groups and then used the marginal effect isolation (MEI) method to predict the remaining 1349 (or 20%) of the genes (Table 1) as described in the Methods section. Self-consistency of predictions on the training set is shown in Table 2. The end result is a clearer understanding of the biological underpinnings of how TFBSs are able to separate the above gene groupings. Because the complete absence of a given TFBS in any of the seven gene types creates a spuriously large and falsely significant Z-score for a given class, we prefiltered the TFBS data to eliminate those that had zero occurrences in any given gene group. BAMarray analysis was then performed on the 216 TFBS that remained after the prefiltering step. Using this approach, we found that the presence of E2FDP1 sites positively influenced the binding of HNF4 in pancreatic islets, while the presence of HNF3 and homeobox sites negatively influenced HNF4 binding. In addition, we found that pairs of HNF4 sites and cooccurring HNF4 and HNF6 sites negatively influence HNF1 binding. A summary of the gene predictions using this method is provided in Table 1. Our analysis of the data produced 1134 correct predictions, or 84% correct (*P* < .0005).

It must be noted that if 100% of the genes were predicted to be unbound, we would have achieved 77% accuracy. However, all of the misclassifications are between the various bound genes and the unbound class, that is, there are no bound genes that were misclassified into other bound categories. Thus, if we excluded genes that were predicted to be unbound, we achieved 98/98 correct predictions. Thus, our estimated false positive rate was zero for bound genes. However, our estimated false negative rate (i.e., predicted unbound genes/total bound genes) was 202/300 or 67%. Thus, while our estimated specificity for bound genes was excellent, our estimated sensitivity (33%) was fairly low. Most difficult to predict were genes bound only in hepatocytes by HNF1 or HNF4, which may be due to influences of newly identified TFBS [16] not included in this analysis, post-translational modifications of HNF1 and/or HNF4, or epigenetic alterations in chromatin structure that differ between pancreatic islets and hepatocytes. Another reason for the low sensitivity is that the vast majority of significant associations of TFBS with HNF1 and HNF4 binding were negative correlations, that is, those factors were *not* likely to bind to promoters containing the significant TFBS.

### 2.2. Ten-Fold Repeated Holdout Training-Test Set Validation

To validate the rules identified by BAMarray analysis of the TFBS patterns, we performed a ten-fold repeated holdout validation of the data. Each group was randomly sampled ten times to split into training and test sets containing 80% or 20% of the data, respectively. Training was performed on a training set of 80% of the genes and prediction was then performed on the remaining test set of 20% of the genes using the ten independent random splits of the data. We expected the BAMarray filtering to be very reproducible according to [17, Theorem 3 and Corollary 1]. The theory indicates that differentially associated factors should be found with probability going to 1 at a rate inversely proportional to the group sample sizes. The outcome of this cross-validation is summarized in Table 4, and the detailed results are given in Supplementary Tables 1–8 (see additional files 10–16: SuppTableS9–S15). In general, the results of the ten random splits of the data were very reproducible, with dozens of sites repeatedly significant in over 50% of the analyses, and many results were reproduced in 100% of the ten random splits. Of great interest was the fact that families of similar TFBS were repeatedly negatively associated with binding of HNF1 or HNF4 in the six classes that we selected (Table 3). For example, V$E2F1, V$E2F1DP2, V$E2F1DP1, V$E2F4DP2, V$E2F1DP1RB, and V$E2F4DP1 were all negatively associated with HNF1 binding in pancreatic islets. In addition, NF*κ*B sites were negatively associated with the three classes of promoters associated with HNF1 binding, while FOX and Homeobox sites were negatively associated with the three classes of promoters associated with HNF4 binding.

To get a better handle on the stability of MEI classification portion of the analysis, we repeated the splitting of the dataset into training and test components 25 times (with the same 80–20 proportions each time). For the MEI classifier, the average total misclassification rate over the 25 splits was 0.1666 with a standard deviation of 0.007. Thus, the overall accuracy over the 25 splits was 83% ± 0.7%. The average false positive rate for bound genes was 0.0099 with a standard deviation of 0.0062. The predictions for each group of genes are summarized in Table 4. Taken as a whole, this repeated splitting exercise indicates that the results presented are highly reproducible.

### 2.3. Contribution of the Large Unbound Class

To gauge the effect of the large unbound class on our sensitivity and false positive rates, we repeated the training and testing of an 80–20 split of the data, this time using only the six bound classes of genes. The results of this classification are shown in Table 5. In general, the maximum false positive rate observed for any class was 16% (HNF4 bound genes in pancreas), and the average false positive rate was 6%. Sensitivity for the six classes ranged from 84% to 100%, while specificity ranged from 95% to 100%. Overall accuracy of the predictions improved to 86% when the unbound class was removed from the dataset. These data suggest that the poor sensitivity that we observed is partially due to the large unbound class of genes from this dataset.

### 2.4. Alternative TFBS Prediction Methods

To compare the performance of the CONFAC approach with other methodologies, we input all of the genes from the HNF ChIP-chip experiment into the oPOSSUM 2.0 software [18, 19]. We performed single site analysis using oPOSSUM and we were able to determine that HNF4 sites were overrepresented in the genes bound by HNF4 and HNF1 in hepatocytes (Table 6). However, since the oPOSSUM 2.0 software only reports the aggregate number of TFBS occurrences in each gene set, and not the individual numbers per gene, it was not possible to use these data for a predictive MEI classification analysis.

However, we did perform additional analyses using two modifications to the CONFAC software. One uses the genomic alignments of human and mouse with a window size of zero, requiring perfect alignment of the sites (Table 7). The second uses the sequences that have significant regulatory potential based on alignment of seven species [20] and uses the window length of 25 bp (Table 8). Using these two approaches, the specificity remains high, and the sensitivity was slightly increased. 

## 3. Discussion

Here we have accurately predicted in vivo occupancy of promoters based on conserved TFBS patterns and public ChIP-chip localization data. The TFBS patterns were generated for the complete set of human RefSeq genes using our CONFAC software, which identifies TFBS that are conserved between human and mouse genomes [1]. A possible reason for our low sensitivity could be the requirement for conservation between human and mouse genomes. However, without this requirement, the gains in sensitivity would likely be lost by losses in specificity. This is supported by an earlier study of 14 gene pairs and 40 verified TFBSs, which found that requiring evolutionary conservation reduced the total number of sites detected by 85% but maintained detection of 83% of verified sites [4]. Thus, comparison of human and mouse genome sequences can greatly reduce the background noise of false positive TFBS with only a small loss in the overall sensitivity for detection of functionally significant TFBS. In addition, analysis of only the bound classes of genes exhibited sensitivity ranging from 86–100%, suggesting that part of the computational challenge lies in the unbalanced nature of these datasets.

While other studies have mined public ChIP-chip data [10] to identify cooccurring TFBS pairs that correlated with expression patterns and localization data using multivariate adaptive regression splines, these studies [14] did not attempt to predict whether a promoter was occupied or unoccupied in a blinded fashion. Our success rate of 84% on an independent test set of 1349 genes demonstrates that given sufficient localization and sequence data, it is possible to separate bound from unbound promoters computationally. One of the challenges with these predictions is the marked lack of balance in the sample sizes between genes bound by a transcription factor and the unbound genes. Since typical approaches tend to minimize overall error rates across all groups, higher error rates on smaller classes are tolerated for smaller error rates on the unbound class. That is, we tend to learn posterior inferences much more accurately on the larger represented class. Under certain situations, this can lead to a bias represented as an underestimation of posterior probabilities of assignment for the smaller classes [21]. There are some approaches to alleviate these biases that deal with this issue in a systematic manner rather than resorting to ad hoc corrections. These approaches include biased sampling in the training set by subsampling from the larger represented group and down weighting observations in the larger represented group [21]. The strategy that we used was to adjust class priors such that posterior class assignments could be made using conventional cutoffs rather than ones that were adjusted for unequal group representation. We will continue to investigate other statistically optimal approaches for bias adjustment with the goal of optimal prediction of TFBS functionality.

Genome-wide analyses of TFBS have been conducted in *Saccharomyces cerevisiae* which have identified combinations of TFBS and integrated the cooccurrence of TFBS in promoter elements with microarray expression data [17, 22, 23]. Bayesian approaches have also been applied to yeast and *C. elegans* [12], but not yet to mammalian genomes, partially because earlier methods for identification of TFBS produced such a high number of false positives. One analysis of the human genome found TFBSs that were enriched in 1 kb of upstream sequences relative to the second exon [24]. This study did find that many transcription factors associated with immune response had TFBS in the promoters of genes annotated for immune system regulation. However, regulatory elements are quite often further than 1 kb upstream of transcription start sites or in intronic sequences, and it is not clear if the second exon of genes represents an optimal background to compare against upstream sequences. Moreover, the study by Long et al. [24] did not use comparative genomics to identify evolutionarily conserved TFBS.

Our approach exhibited high specificity, but low sensitivity for prediction of in vivo binding of HNF4 and HNF1. Part of the reason for the low sensitivity could be due to the nature of the HNF1 binding site, which consists of two 7 bp half-sites with a one-base spacer, resulting in a long (15 bp) PWM [25]. Other approaches for site identification that use half-sites might increase our sensitivity, but CONFAC uses the PWM available in the latest release of MATCH (TRANSFAC Professional Release 11.1). We anticipate that further refinements of our input parameters to include cis-regulatory modules [26] or nucleosome occupancy probabilities [27] may enhance our ability to find positive associations between TFBS sets and in vivo occupancy.

## 4. Conclusions

One of the many challenges of computational biology has been to identify functional genomic binding sites for transcription factors (TFBSs) and the direct downstream targets they affect. Identification of such sites would allow the development of more accurate gene networks and an understanding of important biological pathways. We applied the Conserved Transcription Factor Binding Sites (CONFAC) software to identify evolutionarily conserved and statistically overrepresented TFBS in the region immediately upstream of the complete set of identified human genes. We have developed a novel statistical technique that uses the TFBS patterns to accurately predict binding of transcription factors in a tissue specific manner based on prior biological data. These methods can be applied to additional transcription factors as more biological data becomes available. These methods will allow more accurate predictions of functional transcription factor binding sites, saving time in the wet lab and allowing faster development of more accurate gene networks.

## 5. Methods

### 5.1. CONFAC Analysis

The CONFAC software runs in the linux operating system, using cgi scripts written in the Perl programming language, and accepts lists of genes via a web browser interface (http://confac.emory.edu/). The user inputs a tab-delimited text file containing a unique identifier for the gene name in the first column and a GenBank accession number or RefSeq ID in the second column [1]. The CONFAC software then automatically identifies orthologous murine genes by accessing ortholog lookup tables obtained from the UCSC and ENSEMBL genome databases. The RefSeqs analyzed were based on Tables hg16 refFlat and mm3 refFlat, available at  http://genome.ucsc.edu/cgi-bin/hgTables. The CONFAC analysis was performed using hg16 (July 2003, NCBI Build 34) of the human genome and mm3 (Feb. 2003, NCBI Build 30) of the mouse genome. The settings used for the CONFAC analysis of the complete RefSeq dataset were as follows: matrix similarity: 0.75; core similarity: 0.85; Repeatmasking = ON.

For analysis of using whole genome alignments, the axtNet human-mouse whole genome alignment files (chr[1-22XY].hg[v17].mm[v7].net.axt.gz) were downloaded from the UCSC genome database. Analysis of sequences with positive regulatory potential used the regPotential7X tables also downloaded from the UCSC genome database.

### 5.2. High Throughput Screening with Bayesian ANOVA for Microarrays (BAM)

Bayesian ANOVA was performed using BAMarray 2.0 software available at http://ora.ra.cwru.edu/bamarray/ [28]. BAM is a new statistical technique for detecting differentially expressing genes from multigroup high throughput microarray experiments. The underlying methodology for BAM has been rigorously studied theoretically [15, 29]. BAM is robust to nonnormality of gene expression measurements and to correlations between expression measurements on a given chip [13]. BAM relies on a special type of inferential regularization (i.e., borrowing strength across the data) allowing it to balance the number of false detections against false non-detections hence detecting more genes [13, 29]. This is an oracle (ideal) property guaranteeing lower total gene misclassification [29]. This differs from current statistical methods that protect false detection rates. Controlling false detection rates tends to identify obviously changing genes but it misses many subtle changes. For multigroup designs, BAM adaptively reduces correlations between test statistics on a given gene, enabling signal to be extracted from noise more efficiently, thus allowing true differential gene expression patterns to be readily identified and reducing the number of implausible patterns detected [29].

While originally developed for gene expression microarray experiments, we have extended the usage of BAM for TFBS analysis. Since the BAM methodology does not make parametric assumptions about the nature of the data being analyzed, the count nature of our TFBS data can be handled easily. The number of occurrences of each TFBS for each gene was input into BAMArray. The genes were grouped according to the biological data indicating HNF1 and HNF4 binding, (bound by HNF1 in pancreatic cells, bound by HNF4 in pancreatic cells, bound by HNF1 in hematocytes, bound by HNF4 in hematocytes, bound by HNF1 in both tissues, bound by HNF4 in both tissues, and unbound genes). The baseline group for comparisons was taken to be the class of unbound genes. Analyses were performed using unequal variance between groups and high-accuracy setting. Rare TFBS that had zero conserved occurrences in the groups of genes bound by HNF1 or HNF4 were excluded from the analysis to eliminate spuriously high-negative correlations.

### 5.3. Marginal Effect Isolation (MEI)

Gene predictions were made using a marginal effect isolation (MEI) multigroup classifier. In order to do so, class-specific patterns of differential TFBS associations were pulled from the BAM analysis above. These are patterns that uniquely separate one class from all of the others. These are sometimes termed “hit-and-run” patterns in the original context; they were developed for analyzing differentially expressing genes in multigroup microarray experiments [29]. A simple form of these kinds of patterns can be found in the work of Li and Wong [30], but the BAM-based patterns are much more rigorously defined.

The MEI classifier is a novel multigroup classifier that operates by building separate two group classifiers using these hit-and-run patterns to separate HNF1 and HNF4 bound genes. Specifically, a classifier is built to separate a particular gene set from all of the other by using the hit-and-run pattern TFBS found as predictors and building a two group classifier for discriminating a particular class from all of the others (i.e., the others are put together into a collapsed group). This is repeated for each class one by one except for the baseline group, which is handled separately as described below. For predictions on new test data, these two group classifiers are aggregated in a way that mimics a data-based estimate of a full multigroup Bayes rule. That is, a new observation is tested on each two-group classifier, and is assigned to the group with highest posterior probability of membership. That is, each classifier generates a posterior probability estimate belonging to a group (or not) using only that group's set of hit-and-run TFBS as predictors. A baseline group is always included in the construction of each two-group classifier as part of the collapsed group. If each classifier predicts that the test observation belongs to the collapsed group, then the observation is assigned to the baseline group. The power of this approach for multigroup classification is that it uses specific predictor sets (i.e., TFBS sets) that have been determined by BAM to be particularly informative in uniquely separating individual classes from all of the others. This approach was detailed previously in [31] and is described briefly below.

The test set of TFBS for the genes from the HNF1 and HNF4 in vivo data along with the marginalized TFBS patterns identified by BAM using the training set of HNF1 and HNF4 in vivo data are input for MEI. MEI then outputs its predictions for the test set for which group (HNF1 bound in pancreatic cells, HNF4 bound in pancreatic cells, etc.) each gene should belong to based on its TFBS. The p-value of achieving this number of correct predictions compared to random assignment was based on a chi-squared like test with resampling used to generate a null sampling distribution [32]. 

## Supplementary Material

Supplementary Table S1 presents the complete dataset from the analysis given in Section 2 of the paper. Supplementary Tables S2–S8 show the complete data on conserved TFBS for each of the seven groups analyzed in Section 2.1 of the paper. Supplementary Tables S9–S16 demonstrate detailed results of the cross-validation summarized in Table 4 of the paper.

## Figures and Tables

**Table 1:** Prediction of in vivo occupancy by HNF1 and HNF4. Data from ChIP-chip studies (3) were integrated with CONFAC TFBS data and genes were separated randomly into training and test sets. The BAM MEI classifier was applied to the independent test set of 1349 genes to predict which class each gene belonged based on the TFBS patterns that were predictive of occupancy.

Predicted									
Observed	HNF1 both	HNF1 hep	HNF1 panc	HNF4 both	HNF4 hep	HNF4 panc	Unbound	Total (obs)	Sensitivity
HNF1 both	5	0	0	0	0	0	3	8	63%
HNF1 hep	0	0	0	0	0	0	23	23	0%
HNF1 panc	0	0	3	0	0	0	7	10	30%
HNF4 both	0	0	0	35	0	0	54	89	39%
HNF4 hep	0	0	0	0	14	0	70	84	17%
HNF4 panc	0	0	0	0	0	41	45	86	48%
Unbound	0	0	0	6	5	2	1036	1049	99%
total (pred)	5	0	3	41	19	43	1238	1349	
Specificity	100%	NA	100%	85%	74%	95%	84%		

**Table 2:** Training set self-consistency performance. Data from ChIP-chip studies (3) were integrated with CONFAC TFBS data and genes were separated randomly into training and test sets. The BAM MEI classifier was trained on the training set of 5399 genes and predictions were made on this same set of genes.

Predicted									
Observed	HNF1 both	HNF1 hep	HNF1 panc	HNF4 both	HNF4 hep	HNF4 panc	Unbound	Total (obs)	Sensitivity
HNF1 both	10	0	0	0	0	0	4	14	71%
HNF1 hep	0	4	0	0	0	0	74	78	5%
HNF1 panc	0	0	10	0	0	0	14	24	41%
HNF4 both	0	0	0	130	0	0	142	272	48%
HNF4 hep	0	0	0	0	88	0	295	383	23%
HNF4 panc	0	0	0	0	0	181	159	340	53%
Unbound	0	0	0	6	5	2	4249	4262	99%
total (pred)	10	4	10	136	93	183	4937	5373	
Specificity	100%	NA	100%	89%	85%	96%	86%		

**Table 3:** Rules associated with HNF1 and HNF4 binding identified by 10-fold cross-validation of BAMarray analysis.

TFBS family	Negative association	Positive association
E2F	HNF1-pancreas	HNF4 binding
ETS	HNF1-both	None
MAF	HNF1-hepatocytes	None
NF*κ*B	HNF1-any	None
Homeobox	HNF4-pancreas	None
SOX/TCF	HNF4-both	None
Homeobox	HNF4-hepatocytes	None
FOX/Homeobox	HNF4-any	None

**Table 4:** Summary of MEI predictions from 25 splits of training and test sets. “NA” means that cell could not be calculated for all splits. Otherwise, the means and sd's were calculated from those splits without NA's.

Group	Mean sensitivity (sd)	Mean specificity (sd)
HNF1Both	.252 (.152)	1 (0)
HNF1Hep	0 (0)	NA
HNF1Panc	.352 (.102)	1 (0)
HNF4Both	.388 (.083)	.840 (.023)
HNF4Hep	.130 (.041)	.486 (.201)
HNF4Panc	.430 (.047)	.940 (.070)
Unbound	.982 (.008)	.838 (.004)

**Table 5:** Prediction of in vivo occupancy by HNF1 and HNF4 by BAM MEI analysis after removal of the unbound class from the analysis.

Predicted								
observed	HNF1 both	HNF1 hep	HNF1 panc	HNF4 both	HNF4 hep	HNF4 panc	Total (obs)	Sensitivity
HNF1 both	3	0	0	0	0	0	3	100%
HNF1 hep	0	12	0	0	4	2	18	100%
HNF1 panc	0	0	6	1	2	0	9	100%
HNF4 both	0	0	0	66	3	7	76	93%
HNF4 hep	0	0	0	1	74	9	84	85%
HNF4 panc	0	0	0	4	6	82	92	84%
Total (pred)	3	12	6	72	89	100	282	
Specificity	100%	98%	99%	95%	95%	95%		

**Table 6:** Sites overrepresented by oPOSSUM single site analysis.

Gene set	Significant sites
HNF1-Hepatocytes	HNF4, TCF1
HNF1-Pancreas	None
HNF1-Both	None
HNF4-Hepatocytes	HNF4
HNF4-Pancreas	Staf, GABPA
HNF4-Both	Staf, ELK1, SPIB, Bapx1, ELK4

**Table 7:** Prediction of in vivo occupancy by HNF1 and HNF4 by BAM MEI analysis using human-mouse genomic alignments instead of local pairwise BLAST alignments and a window size of zero.

Predicted									
Observed	HNF1 both	HNF1 hep	HNF1 panc	HNF4 both	HNF4 hep	HNF4 panc	Unbound	Total (obs)	Sensitivity
HNF1 both	3	0	0	0	0	0	1	4	75%
HNF1 hep	0	1	0	0	0	0	18	19	5%
HNF1 panc	0	0	2	0	0	0	4	6	33%
HNF4 both	0	0	0	21	0	0	29	50	42%
HNF4 hep	0	0	0	0	16	0	82	98	16%
HNF4 panc	0	0	0	0	0	34	30	64	53%
Unbound	0	0	0	7	7	1	1259	1274	99%
Total (pred)	3	1	2	28	23	35	1423	1492	
Specificity	100%	100%	100%	75%	67%	95%	88%		

**Table 8:** Prediction of in vivo occupancy by HNF1 and HNF4 by BAM MEI analysis using human-mouse genomic alignments instead of local pairwise BLAST alignments restricted to regions of positive regulatory potential and a window size of 25 bp.

Predicted									
Observed	HNF1 both	HNF1 hep	HNF1 panc	HNF4 both	HNF4 hep	HNF4 panc	Unbound	Total (obs)	Sensitivity
HNF1 both	3	0	0	0	0	0	1	4	75%
HNF1 hep	0	0	0	0	0	0	16	16	0%
HNF1 panc	0	0	1	0	0	0	3	4	25%
HNF4 both	0	0	0	20	0	0	30	50	40%
HNF4 hep	0	0	0	0	18	0	71	89	20%
HNF4 panc	0	0	0	0	0	8	8	16	50%
Unbound	0	0	0	3	3	2	908	916	99%
Total (pred)	3	0	1	23	21	10	1037	1095	
Specificity	100%	NA	100%	87%	86%	80%	87%		

## List of Abbreviations

BAM: Bayesian analysis of microarrays
ChIP-chip: Chromatin immunoprecipitation followed by microarray analysis
CONFAC: Conserved transcription factor binding site software
HNF: Hepatocyte nuclear factor
MEI: Marginal effect isolation
PWM: Position weight matrix
REFSEQ: Reference sequence transcripts
TFBS: Transcription factor binding site.
