# Supplementary material for: Genomic Promoter Analysis Predicts Functional Transcription Factor Binding
Source: Adv Bioinformatics. 2008 Oct 30;2008:369830. doi: 10.1155/2008/369830 (PMC2768302; doi:10.1155/2008/369830)
Supplement: Supplementary file 1 — Supplementary Table S1 presents the complete dataset from the analysis given in Section 2 of the paper. Supplementary Tables S2–S8 show the complete data on conserved TFBS for each of the seven groups analyzed in Section 2.1 of the paper. Supplementary Tables S9–S16 demonstrate detailed results of the cross-validation summarized in Table 4 of the paper. [file 369830.f1.zip › 369830/SuppTable-Legends.doc]

## Supplementary Table Descriptions

# Supplementary Table S1

310 TFBS x 9107 Refseqs

Each column is a TFBS, and each row is a Refseq gene. The annotation columns are the HUGO Symbol and Refseq ID. The number in each cell is the number of conserved occurrences for that TFBS between human and mouse genomes.

# Supplementary Table S2

258 TFBS x 5270 Refseqs (Unbound Group)

Each column is a TFBS, and each row is a Refseq gene. HUGO Symbol, Refseq ID, Bound (bound in any ChIP-chip experiment), HNF1-hep (1= bound by HNF1 in hepatocytes), HNF1-panc (1=bound by HNF1 in pancreatic islets), HNF4-hep (1=bound by HNF4 in hepatocytes), and HNF4-panc (1=bound by HNF4 in pancreatic islets). The number in each cell is the number of conserved occurrences for that TFBS between human and mouse genomes.

# Supplementary Table S3

258 TFBS x 103 Refseqs (HNF1-Hep Group)

Each column is a TFBS, and each row is a Refseq gene. The number in each cell is the number of conserved occurrences for that TFBS between human and mouse genomes. Annotation colunns are as in Supplementary Table S2.

# Supplementary Table S4

258 TFBS x 23 Refseqs (HNF1-Both Group)

Each column is a TFBS, and each row is a Refseq gene. The number in each cell is the number of conserved occurrences for that TFBS between human and mouse genomes. Annotation colunns are as in Supplementary Table S2.

# Supplementary Table S5

258 TFBS x 35 Refseqs (HNF1-Panc Group)

Each column is a TFBS, and each row is a Refseq gene. The number in each cell is the number of conserved occurrences for that TFBS between human and mouse genomes. Annotation colunns are as in Supplementary Table S2.

# Supplementary Table S6

258 TFBS x 528 Refseqs (HNF4-Hep Group)

Each column is a TFBS, and each row is a Refseq gene. The number in each cell is the number of conserved occurrences for that TFBS between human and mouse genomes. Annotation colunns are as in Supplementary Table S2.

# Supplementary Table S7

258 TFBS x 409 Refseqs (HNF4-Both Group)

Each column is a TFBS, and each row is a Refseq gene. The number in each cell is the number of conserved occurrences for that TFBS between human and mouse genomes. Annotation colunns are as in Supplementary Table S2.

# Supplementary Table S8

258 TFBS x 436 Refseqs (HNF4-Panc Group)

Each column is a TFBS, and each row is a Refseq gene. The number in each cell is the number of conserved occurrences for that TFBS between human and mouse genomes. Annotation colunns are as in Supplementary Table S2.

**Supplementary Table S9:** TFBS Significantly associated with HNF1-Binding in Pancreatic islets. *Num-Splits-Sig* is the number of the 10 random splits in which the TFBS was found to be significant for any group, and the *% significant* is the percentage of those splits. *Hit-and-run* is the number of splits for which the TFBS was significant for only a single group and *% unique hit-and-run* is the percentage of runs for which the TFBS was significant that it was also a hit-and-run TFBS. *HNF1-Panc* is the number of splits for which the TFBS was significantly associated with the HNF1-Panc group. Negative numbers indicate negative association (i.e. the presence of the TFBS is correlated with the absence of HNF1 binding). *%HNF1-Panc* is the percentage of significant splits that the TFBS was also significant for the HNF1-Panc group.

**Supplementary Table S10:** TFBS Significantly associated with HNF1-Binding in Pancreas and Hepatocytes. *Num-Splits-Sig* is the number of the 10 random splits in which the TFBS was found to be significant for any group, and the *% significant* is the percentage of those splits.  *Hit-and-run* is the number of splits for which the TFBS was significant for only a single group and *% unique hit-and-run* is the percentage of runs for which the TFBS was significant that it was also a hit-and-run TFBS. *HNF1-both* is the number of splits for which the TFBS was significantly associated with the HNF1-both group. Negative numbers indicate negative association (i.e. the presence of the TFBS is correlated with the absence of HNF1 binding). *%HNF1-both* is the percentage of significant splits that the TFBS was also significant for the HNF1-both group.

**Supplementary Table S11:** TFBS Significantly associated with HNF1-Binding in Hepatocytes. *Num-Splits-Sig* is the number of the 10 random splits in which the TFBS was found to be significant for any group, and the *% significant* is the percentage of those splits.  *Hit-and-run* is the number of splits for which the TFBS was significant for only a single group and *% unique hit-and-run* is the percentage of runs for which the TFBS was significant that it was also a hit-and-run TFBS. *HNF1-Hep* is the number of splits for which the TFBS was significantly associated with the HNF1-Hep group. Negative numbers indicate negative association (i.e. the presence of the TFBS is correlated with the absence of HNF1 binding). *%HNF1-Hep* is the percentage of significant splits that the TFBS was also significant for the HNF1-Hep group.

**Supplementary Table S12:** TFBS Significantly associated with HNF4-Binding in Pancreatic islets. *Num-Splits-Sig* is the number of the 10 random splits in which the TFBS was found to be significant for any group, and the *% significant* is the percentage of those splits.  *Hit-and-run* is the number of splits for which the TFBS was significant for only a single group and *% unique hit-and-run* is the percentage of runs for which the TFBS was significant that it was also a hit-and-run TFBS. *HNF4-Panc* is the number of splits for which the TFBS was significantly associated with the HNF4-Panc group. Negative numbers indicate negative association (i.e. the presence of the TFBS is correlated with the absence of HNF4 binding). *%HNF4-Panc* is the percentage of significant splits that the TFBS was also significant for the HNF4-Panc group.

**Supplementary Table S13:** TFBS Significantly associated with HNF4-Binding in Pancreatic islets and Hepatocytes. TFBS Significantly associated with HNF4-Binding in Pancreas and Hepatocytes. *Num-Splits-Sig* is the number of the 10 random splits in which the TFBS was found to be significant for any group, and the *% significant* is the percentage of those splits.  *Hit-and-run* is the number of splits for which the TFBS was significant for only a single group and *% unique hit-and-run* is the percentage of runs for which the TFBS was significant that it was also a hit-and-run TFBS. *HNF4-both* is the number of splits for which the TFBS was significantly associated with the HNF4-both group. Negative numbers indicate negative association (i.e. the presence of the TFBS is correlated with the absence of HNF4 binding). *%HNF4-both* is the percentage of significant splits that the TFBS was also significant for the HNF4-both group.

**Supplementary Table S14:** TFBS Significantly associated with HNF4-Binding in Hepatocytes. *Num-Splits-Sig* is the number of the 10 random splits in which the TFBS was found to be significant for any group, and the *% significant* is the percentage of those splits.  *Hit-and-run* is the number of splits for which the TFBS was significant for only a single group and *% unique hit-and-run* is the percentage of runs for which the TFBS was significant that it was also a hit-and-run TFBS. *HNF4-Hep* is the number of splits for which the TFBS was significantly associated with the HNF4-Hep group. Negative numbers indicate negative association (i.e. the presence of the TFBS is correlated with the absence of HNF4 binding). *%HNF4-Hep* is the percentage of significant splits that the TFBS was also significant for the HNF4-Hep group.

**Supplementary Table S15:** TFBS Significantly associated with HNF4-Binding. These TFBS were significantly associated with multiple HNF4 bound groups. The theoretical maximum is 30, which corresponds to TFBS that are significant for all three groups (HNF4-Panc, HNF4-Hep, HNF4-Both) in all ten splits. The bottom two rows indicate the positive association of E2F sites with HNF4 binding.

**Supplementary Table S16:** TFBS Significantly associated with HNF1-Binding. These TFBS were significantly associated with multiple HNF1 bound groups. The theoretical maximum is 30, which corresponds to TFBS that are significant for all three groups (HNF1-Panc, HNF1-Hep, HNF1-Both) in all ten splits.
